# A care bundle added to standard care versus standard care for the prevention of surgical site infections after abdominal surgery (EPO_2_CH trial): a randomised, open label, pragmatic, superiority multicentre trial

**DOI:** 10.1016/j.lanepe.2025.101448

**Published:** 2025-09-17

**Authors:** Niels Wolfhagen, Quirine J.J. Boldingh, Wouter J. Bom, Linda M. Posthuma, Jochem C.G. Scheijmans, Bart M.F. van der Leeuw, Joost A.B. van der Hoeven, Jens-Peter Hering, Dirk J.A. Sonneveld, Otto E. van Geffen, Eduard R. Hendriks, Ewoud B. Kluyver, Ahmet Demirkiran, Luc R.C.W. van Lonkhuijzen, Marcel G.W. Dijkgraaf, Markus W. Hollmann, Marja A. Boermeester, Stijn W. de Jonge

**Affiliations:** a Department of Surgery, Amsterdam UMC Location University of Amsterdam, the Netherlands; b Amsterdam Gastroenterology, Endocrinology and Metabolism, Amsterdam, the Netherlands; c Department of Surgery, Dijklander Ziekenhuis, Hoorn, the Netherlands; d Department of Anaesthesiology, Amsterdam UMC, University of Amsterdam, Amsterdam, the Netherlands; e Department of Anaesthesiology, Albert Schweitzer Hospital, Dordrecht, the Netherlands; f Department of Surgery, Albert Schweitzer Hospital, Dordrecht, the Netherlands; g Department of Anaesthesiology, Dijklander Ziekenhuis, Hoorn, the Netherlands; h Department of Anaesthesiology, Tergooi MC, Hilversum, the Netherlands; i Department of Surgery, Tergooi MC, Hilversum, the Netherlands; j Department of Anaesthesiology, Rode Kruis Hospital, the Netherlands; k Department of Surgery, Rode Kruis Hospital, the Netherlands; l Department of Gynaecologic Oncology, Centre for Gynaecologic Oncology Amsterdam, Amsterdam UMC, University of Amsterdam, Amsterdam, the Netherlands; m Amsterdam UMC Location University of Amsterdam, Epidemiology and Data Science, Amsterdam, the Netherlands; n Amsterdam Public Health, Methodology, Amsterdam, the Netherlands

**Keywords:** Surgical site infections, Hospital acquired infections, Surgery, Anaesthesia, Prevention

## Abstract

**Background:**

Surgical site infections (SSI) are common. We selected five interventions from recent SSI prevention guidelines, to form the Enhanced PeriOperative Care and Health program (EPO_2_CH), a perioperative care bundle. We aimed to investigate the effect of the EPO_2_CH bundle on the incidence of SSI.

**Methods:**

The EPO_2_CH trial concerns an open label, pragmatic, randomised controlled parallel-group multicentre trial, in which we assigned patients, scheduled for elective abdominal surgery with incisions larger than five centimetres, to either standard care or standard care plus the EPO_2_CH bundle consisting of intraoperative high fraction of inspired oxygen; Goal-Directed Fluid Therapy; normothermia; perioperative glucose control; and incisional wound irrigation. The study was conducted in seven hospitals in the Netherlands. Patients were randomised per hospital per day in a 1:1 ratio with variable block sizes using an internet-based automated assignment system. The primary outcome was the incidence of SSI within 30 days in the intention-to-treat population. This study is registered at CCMO register (NL-OMON50566).

**Findings:**

Between March 1st, 2016, and March 26th, 2020, 1777 patients were included. The intervention group included 869 patients (mean age 63.1, 467 female and 402 male) versus 908 in the control group (mean age 64.0, 530 female and 378 male). The incidence of SSI was 18.4% (160/869) in the intervention and 18.9% (172/908) in the control group; relative risk 0.98 (95% CI: 0.81–1.18) in the intention-to-treat analysis and 0.91 (95% CI: 0.60–1.37) in the per-protocol analysis. The percentage of patients with a serious adverse event was 33.3% (289/869) versus 33.5% (304/908), RR 0.99, 95% CI 0.87–1.23.

**Interpretation:**

In a high-income health care setting, a care bundle did not lead to a lower incidence of surgical site infections when added to standard care including preoperative systemic antibiotic prophylaxis and alcohol-based surgical skin preparation. Considering the persistent high risk of SSI, research into interventions that may help to reduce this risk remains urgently needed.

**Funding:**

The 10.13039/501100001826Netherlands Organisation for Health Research and Development (ZonMW), and co-financed by 10.13039/501100009248Innovatiefonds Zorgverzekeraars, and 10.13039/100009933Ethicon.

**Research in context**

**Evidence before this study**

We previously (inception until June 6th, 2020) searched MEDLINE (PubMed), Excerpta Medica Database (Embase), Cumulative Index to Nursing and Allied Health Literature (CINAHL), and the Cochrane Central Register of Controlled Trials (CENTRAL) for evidence on the effect of care bundles to help prevent SSI using an extensive search strategy including the following search terms and relevant variations: “surgical site infection”, “bundles”, “package”, “checklist”, “tool”, “pathway”, “improvement”, “implement”, “strategy”, “Evidence-Based”, “SCIP”, “NSQIP” without any language or publication data restrictions. Four identified randomised controlled trials were too heterogeneous for pooled meta-analysis. Meta-analysis of 13 interrupted time series suggested perioperative care bundles help prevent SSI [Level change −1.16 (95% CI -1.78 to −0.53)]. Overall, the studies were of limited quality with considerable risk of bias. On March 27th 2025, we updated the search and no new relevant RCTs were identified. The effect of an evidence-based care bundle to help prevent SSI remains uncertain.

**Added value of this study**

This randomised controlled trial showed that, in a high-income setting, a care bundle comprising of evidence-based interventions extracted from recent including normothermia, normoglycemia, Goal-Directed Fluid Therapy, hyperoxygenation, and wound irrigation did not lead to a lower incidence of surgical site infections among patients undergoing elective abdominal surgery when added to standard care comprising measures such as timely preoperative systemic antibiotic prophylaxis, avoidance of hair removal, and alcohol-based surgical skin preparation. This is the largest randomised controlled trial that studies a perioperative care bundle based on five interventions which are individually supported by evidence from meta-analysis and international guidelines. We found that SSI incidence remains high and that a bundle of evidence-based interventions extracted from recent guidelines could not reduce this risk.

**Implications of all the available evidence**

Given the recent guideline recommendations and efforts to implement these globally, these findings have important practice and policy implications. Resources should be allocated towards implementation of interventions that have been proven beyond any doubt such as hand hygiene, surgical site preparation and adequate use of preoperative antibiotic prophylaxis. Considering the persistent high risk of SSI even in high resource settings, research into interventions that may help reduce this risk remains urgently needed.

## Introduction

Surgical site infections (SSI) cause excess morbidity, mortality, and increased costs.^1^ A large proportion is deemed preventable with evidence-based strategies,^2^ but despite efforts employing individual interventions, no major progression has been made. In management of sepsis and prevention of catheter-related bloodstream infections, systematic approaches or care bundles have been proven effective.^3^, ^4^, ^5^ Evidence on the effect of care bundles to reduce SSI is limited.^6^ A meta-analysis investigating care bundles used to reduce SSI showed large variation and inconsistency in the effect.^6^

The World Health Organization (WHO), the Centers for Disease Control and prevention (CDC), and the National Institute for Health and Clinical Excellence (NICE) issued guidelines for the prevention of SSI.^7^, ^8^, ^9^, ^10^ These highlight that care can be optimised beyond current standards. We selected five readily available, inexpensive and easy to deliver interventions from these guidelines, to form the Enhanced PeriOperative Care and Health program (EPO_2_CH), a perioperative care bundle aiming to reduce the risk of SSI and designed a multicentre, randomised controlled trial. We aim to test the hypothesis that the EPO_2_CH bundle added to standard care would reduce the incidence of SSI within 30 days after surgery.

## Methods

### Study design

The EPO_2_CH trial (NL-OMON50566) is an investigator-initiated open label, pragmatic, randomised controlled parallel-group multicentre superiority trial that took place in seven hospitals in the Netherlands. The Amsterdam UMC Medical Ethics Committee (ref. 2015_121) approved the trial on July 22nd 2015 Local review boards at participating centres also approved the trial. Details of the design have been published.^11^^,^^12^ An independent Data Safety Monitoring Board (DSMB) monitored safety. The first two and last four authors had full access to the data. The first and last author drafted the manuscript, and all authors critically reviewed it.

### Participants

Patients, 18 years of age or older, scheduled for elective open or laparoscopic abdominal surgery with an incision larger than five centimetres were eligible. Emergency surgery, two-staged procedures, and reoperations after recent surgery outside the study were not eligible. Patients that were unable to read or understand the informed consent material, pregnant, or included in a conflicting trial were ineligible. If intraoperative findings affected the treatment plan resulting ineligibility, participants were excluded and replaced. Participants provided written informed consent either preoperatively in the outpatient department or preoperatively during admission. Sex was self-reported by participants, options were male, female, other. To our knowledge, there is no literature or biological argument that would suggest that race or ethnicity has an independent association with SSI, therefore no data regarding ethnicity was collected.

### Randomisation and masking

Central random treatment allocation was performed per operating day to limit contamination between consecutive procedures by the same team. Numerous OR teams were involved, limiting contamination. Days were randomised in a 1:1 ratio according to variable block sizes, stratified per trial site, using an internet based automated assignment system (CASTOR EDC^13^) to either the EPO_2_CH bundle plus standard care or standard care alone.

### Procedures

The *EPO*_*2*_*CH bundle* comprises of (1) intraoperative administration of 80% fraction of inspired oxygen (FiO_2_) in the period after intubation until extubation, (2) Goal-Directed Fluid Therapy (GDFT) based on dynamic preload including pulse pressure variation or systolic pressure variation when available or by compensating hypotensive periods with vasopressors until a maximum of 0.08 μg per kilogram per minute before fluids are administered, (3) active warming with a hot air blanket, or if unavailable with preheated blankets, from arrival at the holding area until 2 h after surgery to maintain core temperature above 36.5 °C, (4) perioperative glucose control (in diabetics and non-diabetics) from start surgery (every hour during surgery, once in the recovery)until the second postoperative day (daily (unfastened)), and administration of insulin with subsequent control of its effect in case of glucose above 10 mmol per litre (dose and agent at the treating physicians discretion), (5) incisional wound irrigation with an aqueous antiseptic agent (0.35%–10% aqueous povidone iodine solution or 1% aqueous chlorhexidine gluconate) before closure followed by lavage with saline. The volume of antiseptic and dwell time was left at the surgeons’ discretion. No intra-abdominal lavage was performed. In case of laparoscopic procedures, the wound used for extraction of the surgical specimen was irrigated.

*Standard care* in this high-income health care setting typically included 30–40% FiO_2_, hemodynamic management based on clinical judgment, active intraoperative warming but no pre- or postoperative warming, glucose control only in diabetic patients, and no incisional wound irrigation. To avoid withholding good care in the control arm, surgeons and anaesthesiologists were permitted to diverge from standard care at their discretion.

To monitor compliance, the treating anaesthesiologist and surgeon filled in a postoperative survey after each procedure on the execution of the interventions. Additionally, process measures, taken from the hospital information system, if available, were used to track success of these interventions. Standard SSI prevention measures included: preoperative systemic antibiotic prophylaxis (SAP) consisting of 2 g cefazoline and 0.5 g metronidazole when indicated within 60 to 15 min prior to incision, and repeated in case of long procedures or excessive blood loss, avoidance of preoperative hair removal (clipper used if indicated), sterile technique, hand hygiene according to standards, and surgical site preparation with alcohol-based povidone or chlorhexidine gluconate.

### Outcomes

The primary outcome was the incidence of SSI as defined by the Centers for Disease Control and prevention (CDC) within 30 days.^14^ Secondary outcomes included, amongst others, type of surgical site infection, incidence of anastomotic leakage of functional anastomosis (i.e., anastomosis without a diversion ostomy) after 30 days, SSI incidence within 90 days, length of hospital stay, ICU admissions, hospital readmission rate, Serious Adverse Events (SAE) according to the Clavien Dindo classification, health utility, disability, costs, and SSI follow-up with a self-assessment questionnaire and self-reported wound photos. Due to the inherent risk of mild adverse events after major abdominal surgery and the diversity of the intervention after consultation of the DSMB and the Ethical Review Committee, mild adverse events including Clavien Dindo category I or II were not reported. SAE were categorized according to MedDRA classification.^15^ Analysis of other secondary outcomes such as health utility and disability, costs and long term follow up after one year will be reported separately. Health scores and costs will be reported according to primary study outcomes. Analysis of self-assessment questionnaires is not reported here as no formal process of translation and validation was performed. Routine postoperative clinical care and follow typically constituted of daily judgement of abdominal wound during admission, a two-to-three-week postoperative visit at the outpatient clinic including wound evaluation and any additional evaluation in case of concerns. No additional follow up visit at the end of the study was planned. Patients were explicitly instructed to contact their clinicians in case of any wound problems, when they did not, we assumed they did not develop an SSI. Outcomes were extracted from registered complications with parallel assessment through medical chart review by the trial management team. Outcome assessors, ward doctors and surgeons responsible for complication registry, were not involved in execution of the interventions and will be kept unaware of treatment allocation. Outcomes and methods of data collection are described in detail in the protocol.^11^

### Statistical analyses

Details of the statistical analysis plan were previously published.^11^^,^^12^ The estimated SSI incidence was 9.1% with standard care. To detect a 30% relative risk reduction with 80% power and a 5% level of significance, the sample size was 3000 participants, 1500 in each arm. The DSMB conducted an a priori planned evaluation of the sample size assumptions after follow-up of 1500 participants and detected an SSI incidence of 19.3% in the control group resulting in a revised sample size of 1216 participants. At this time already 1777 patients had been included.^12^

In case of missing outcome data, participants would be considered lost to follow up for the respective outcome. We assumed this to be unrelated to the outcome and conducted a complete case analysis. We conducted the primary analysis according to the intention to treat principle. Patients were not excluded from the analysis in case of reoperation within the follow up period because reoperation is a common downstream consequence of the primary outcome and only incidental cases of reoperation for other causes within the follow up are expected. Dichotomous outcomes were analysed using a log-binomial model, estimating relative risks with corresponding confidence intervals. Intention-to-treat analysis included a covariate for hospital type to account for stratification using a fixed effect. Poisson regression was used to test for differences in length of stay.

Per-protocol analyses were conducted according to predefined process measures for success of these interventions and to the treatment received as recorded in postoperative surveys. In the control group any temperature management was considered per protocol due to the pragmatic nature of the trial. For the per-protocol analyses, VanderWeele and Shpitser’s principles of confounder selection were applied and variables were selected using backward selection.^16^ In groups of 100 consecutive participants, ANOVA and Chi^2^ tests were conducted to analyse if the standard care in the control group changed over time. Pearson correlation coefficient was calculated to determine correlation between the trial progression and the use of the interventions in the control group. Attributive effect of each individual intervention was analysed including two- and three-way interactions of hyperoxygenation, GDFT and normothermia. Sex is not considered a confounder or effect modifier; analyses were not stratified for sex. Analyses were conducted using R version 4.2.1 [R Foundation for Statistical Computing, Vienna, Austria].

### Role of the funding source

The funder of the study had no role in study design, data collection, data analysis, data interpretation, or writing of the report.

## Results

**Fig. 1:**
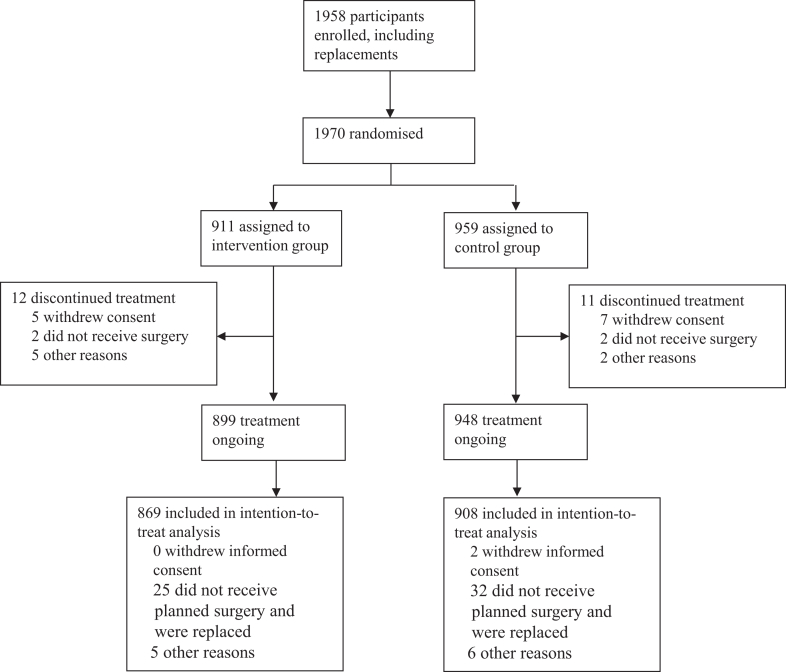
EPO_2_CH trial flow diagram.

From March 1st, 2016, to March 26th, 2020, 1870 patients were randomised. Follow up was completed by June 24th, 2020. In total, 1777 patients were included in the analysis: 869 participants in the intervention group versus 908 participants in the control group. Reasons for exclusions from the analysis are depicted in Fig. 1. Patients that did discontinued treatment were either because they withdrew consent between inclusion and surgery, or the surgery was changed so that patients did not meet inclusion criteria anymore. No patients were considered as lost to follow up. Randomisation per operating day resulted in a slight difference in group size. A total of 774 days were randomised with a mean number of participants of 2.34 ± 1.35 participants per day. One academic hospital, three top clinical hospitals and three general teaching hospitals participated.

Baseline and procedure characteristics were comparable between both treatment groups (Tables 1 and 2). Average administered crystalloid and colloid fluid volumes during surgery were 1620 ± 1160 ml and 242 ± 417 ml, respectively, in the intervention group versus 1490 ± 1010 ml and 176 ± 383 ml in the control group. Preoperative antibiotic prophylaxis was administered within 15–60 min preoperatively in 92.6% (789/869) in the intervention group versus 92.7% (840/908) in the control group with. 2 g Cefazoline and 0.5 g metronidazole as standard regimen. Specific agents administered are detailed in Online Appendix 1. All wounds were primarily closed.

**Table 1:** Baseline characteristics.

Characteristic	Intervention group (n = 869)	Control group (n = 908)
Sex, female	467 (53.7%)	530 (58.4%)
Sex, male	402 (46.3%)	378 (41.6%)
Age, years	63.1 (12.9)	64.0 (12.4)
BMI, kg/m^2^	26.6 (4.70)	26.4 (5.24)
Missing	0	3 (0.3%)
ASA physical status score		
ASA I	91 (10.5%)	108 (11.9%)
ASA II	563 (65.2%)	583 (64.5%)
ASA III	203 (23.5%)	206 (22.8%)
ASA IV	7 (0.810%)	7 (0.774%)
Missing	5 (0.6%)	2 (0.2%)
Smoking	350 (40.6%)	365 (40.4%)
Missing	6 (0.7%)	4 (0.4%)
Diabetes mellitus	141 (16.2%)	126 (13.9%)
Chronic Obstructive Pulmonary Disease	70 (8.06%)	63 (6.94%)
History of abdominal surgery	415 (47.8%)	440 (48.5%)
Cardiovascular disease excl. hypertension	141 (16.2%)	158 (17.4%)
Type of hospital		
Academic	436 (50.2%)	461 (50.8%)
Top-clinical	246 (28.3%)	254 (28.0%)
General	187 (21.5%)	193 (21.3%)
Indication for surgery		
Benign	234 (26.9%)	224 (24.7%)
Malignancy	635 (73.1%)	683 (75.2%)
Missing	0	1 (0.1%)
Surgery type		
General surgery	126 (14.5%)	110 (12.1%)
Upper gastrointestinal surgery	23 (2.65%)	24 (2.65%)
Hepato-pancreatico-biliary	132 (15.2%)	141 (15.5%)
Colorectal surgery	469 (54.0%)	513 (56.5%)
Gynecologic surgery	119 (13.7%)	120 (13.2%)

Continuous variables are expressed as mean (SD), discrete variables are expressed as number (%).

Sex was self-reported by participants, options were male, female, other.

**Table 2:** Perioperative procedure characteristics.

	Intervention group (n = 869)	Control group (n = 908)
Preoperative antibiotics *within 15 – 60 min before incision*	798 (92.6%)	840 (92.7%)
Missing	7 (0.8%)	2 (0.2%)
Procedure duration, hours	3.28 (1.67)	3.17 (1.60)
Missing	2 (0.2%)	1 (0.1%)
Mean arterial pressure, mmHg	81.5 (11.3)	79.7 (9.7)
Missing	100 (11.5%)	107 (11.8%)
Heart rate, bpm	67.8 (10.7)	66.7 (10.5)
Missing	98 (9.8%)	89 (9.8%)
Intraoperative core temperature, °C	36.4 (0.4)	36.3 (0.4)
Missing	153 (16.9%)	152 (17.5%)
Oxygen saturation	99.5 (0.810)	99.1 (1.15)
Missing	91 (10.5%)	92 (10.1%)
Estimated blood loss, ml	414 (771)	356 (603)
Missing	113 (13.0%)	111 (12.2%)
Crystalloid infusion, ml	1620 (1160)	1490 (1010)
Missing	1 (0.1%)	3 (0.3%)
Colloid infusion, ml	242 (417)	176 (383)
Missing	7 (0.8%)	5 (0.6%)
Patients receiving blood products	35 (4.0%)	37 (4.1%)
Missing	1 (0.1%)	1 (0.1%)
Noradrenaline, ug	721 (1070)	672 (1050)
Missing	188 (21.6%)	199 (21.9%)
Noradrenaline per kg per hour, ug	2.85 (6.60)	2.47 (5.00)
Missing	191 (22.0%)	204 (22.5%)
Fenylefrine, ug	213 (659)	320 (864)
Missing	100 (11.5%)	110 (12.1%)
Fenylefrine per kg per hour, ug	0.939 (2.68)	1.71 (4.75)
Missing	101 (11.6%)	113 (12.4%)
Efedrine, mg	8.13 (9.67)	9.11 (10.6)
Missing	43 (4.9%)	37 (4.1%)
Efedrine per kg per hour, mg	0.037 (0.052)	0.048 (0.064)
Missing	43 (4.9%)	37 (4.1%)
Postoperative pain, Mean maximum VAS at day of surgery	3.79 (1.94)	3.81 (1.96)
Missing	164 (18.9%)	179 (19.7%)
Postoperative pain, Sum VAS postoperative day 1–3	23.5 (19.9)	22.6 (17.2)
Missing	101 (11.6%)	104 (11.5%)
Epidural anaesthesia	351 (40.4%)	390 (43.0%)
Wound Class (CDC)		
1	62 (7.1%)	40 (4.4%)
2	759 (87.3%)	823 (90.6%)
3	30 (3.5%)	29 (3.2%)
4	18 (2.1%)	16 (1.8%)
Functional anastomosis	531 (61.6%)	566 (62.8%)

Continuous variables are expressed as mean (SD), discrete variables are expressed as number (%).

The incidence of SSI within 30 days in the intervention group was 18.4% (160/869) compared to 18.9% (172/908) in the control group; risk 0.98 (95% CI: 0.81–1.18). Incidences of superficial, deep, and organ-space were comparable (Table 3). The incidence of anastomotic leakage within 30 days was 9.2% (49/531) in the intervention and 10.9% (62/566) in the control group (RR 0.81 (95% CI: 0.55–1.11)). Details regarding the treatment of SSI are outlined in Online Appendix 2.

**Table 3:** Primary and secondary clinical outcomes.

	Intervention group (n = 869)	Control group (n = 908)	Relative Risk (95% CI) ITT (n = 1777)	Intervention group (n = 145)	Control group (n = 444)	Adjusted relative risk (95% CI) PP (n = 589)^a^
Surgical site infection, 30 days	160 (18.4%)	172 (18.9%)	0.98 (0.81–1.18)	23 (15.9%)	67 (15.1%)	0.91 (0.60–1.37)
Superficial	52 (6.0%)	52 (5.7%)	1.04 (0.72–1.51)	6 (4.1%)	30 (6.8%)	0.51 (0.21–1.21)
Deep/Organ space (including anastomotic leakage)	107 (12.3%)	119 (13.1%)	0.95 (0.74–1.21)	17 (11.7%)	36 (8.1%)	1.52 (0.89–2.60)^b^
Anastomotic leakage, 30 days	49/531 (9.2%)^c^	62/566 (10.9%)^c^	0.81 (0.55–1.11)	11/107 (10.3%)^c^	18/261 (6.9%)^c^	1.52 (0.73–3.61)
Surgical site infection, 90 days	177 (20.4%)	182 (20.0%)	1.02 (0.85–1.23)	28 (19.3%)	71 (16.1%)	1.11 (0.76–1.61)
Length of stay, median (IQR)	6 (4–9)	6 (4–9)	0.99 (0.96–1.02)^e^	6 (4–9)	6 (4–9)	1.24 (1.16–1.32)^d^

a Adjusted for cardiovascular disease, COPD, insulin dependent diabetes, OR duration and type of surgery.

b Adjusted for COPD, insulin dependent diabetes, OR duration and type of surgery.

c Denominator refers to the patients at risk of anastomotic leakage.

d Poisson regression analysis.

e n = 1752.

Execution of the intervention as reported in postoperative surveys is described in Online Appendix 3. In the intervention group, active care for pre- or postoperative warming was provided in 84.5% (652/772) versus 20.5% (165/803) in the control group, GDFT in 84.8% (669/789) in the intervention group versus 21.2% (167/788) in the control group, intraoperative glucose measurement in 93.3% (603/646) versus 21.0% (120/747), and wound irrigation in 89.8% (711/792) versus 17.0% (141/830) respectively. Details regarding wound irrigation are provided in Online Appendix 4. Compliance to hyperoxygenation was solely based on process parameters.

We formulated strict definitions for successful execution of the predefined EPO_2_CH interventions based on verifiable process measures for normothermia, normoglycemia, and hyperoxygenation.^12^ These definitions are provided in Online Appendix 5 and the results are described in Online Appendix 6. Participants may have met criteria for an intervention after receiving standard care (e.g., glucose monitoring due to diabetes). Normothermia according to protocol definitions was achieved in 61.0% (530/869) in the intervention group versus 49.4% (449/908) in the control group, glucose control in 58.2% (506/869) in the intervention group versus 5.5% (50/908) in the control group. Hyperoxygenation was achieved in 75.8% (659/869) in the intervention group with an average FiO_2_ of 74.4% ± 10.3 versus 4.7% (43/908) in the control group with an average FiO_2_ of 48.6% ± 10.5.

Some interventions such as active warming, hyperoxygenation and glucose control have measurable consequences on the participants. The mean intraoperative minimum temperature was 35.5 ± 1.1 °C in the intervention group versus 35.6 ± 1.4 °C in the control group, the mean temperature at the PACU 36.8 ± 0.6 °C versus 36.6 ± 0.6 °C. The mean measured FiO_2_ in the intervention group was 74.4 ± 10.3% versus 48.6 ± 10.5% in the control group. With active monitoring 14.2% (123/869) of patients in the intervention group were found to have glucose levels above 10 mmol per litre while in 13.6% (118/869) insulin was administered compared to 7.2% (65/908) and 5.2% (47/908) in the control group respectively. Almost half of these patients in the intervention group did not have diabetes. Details regarding glucose measurement are provided in Online Appendix 7.

The per-protocol analysis included all participants from the intervention group in which *all* five interventions were perfectly executed (n = 145) versus participants in the control group who did not receive any of the individual interventions (n = 444). Confounder adjusted relative risk for SSI within 30 days for the intervention group compared to the control group was RR 0.91 (95% CI: 0.60–1.37). Details on confounder selection are described in Online Appendix 8. Relative risk for readmission was 1.67 (95% CI: 0.95–2.95) and for ICU admission 1.45 (95% CI: 0.85–2.50).

Safety outcomes were comparable in both groups, Table 4. The incidence of readmission was 9.8% (85/869) in the intervention group and 8.4% (76/908) in the control group (estimated RR 1.17, 95% CI: 0.87–1.57). The number of patients with one or multiple SAEs was comparable between the two groups. A total of 289 patients had one or multiple SAEs [total of 531 SAEs] of which 108 patients required either a radiological or surgical intervention in the intervention group versus 304 patients [total of 543 SAEs] in the control group of which 129 patients required an intervention. Most common type of SAEs were categorized as gastrointestinal disorders (30.3% (161/531) versus 33.3% (181/543) in the control group). In the intervention group seven patients required reoperation for other reasons than SSI versus five in the control group. More detail regarding SAEs is provided in Online Appendix 7. SAE resulting into mortality (Clavien Dindo V) was rare but occurred more frequently in the intervention group (1.7% (15/869) versus 0.9% (8/908)). After review of each case, the DSMB concluded none were related to the interventions. No patient was withdrawn from the trial due to safety reasons. Safety analyses were similar in the safety population, Online Appendix 9.

**Table 4:** Safety outcomes.

	Intervention group (n = 869)	Control group (n = 908)	Relative Risk (95% CI) ITT (n = 1777)	Intervention group (n = 145)	Control group (n = 444)	Adjusted relative risk (95% CI) PP (n = 589)^a^
Readmissions—yes/no	85 (9.8%)	76 (8.4%)	1.17 (0.87–1.57)	17 (11.7%)	29 (6.5%)	1.67 (0.95–2.95)
ICU admission—yes/no	81 (9.3%)	92 (10.1%)	0.91 (0.69–1.22)	16 (11.0%)	32 (7.2%)	1.45 (0.85–2.50)
Serious adverse events (any), No patients (%)	289 (33.3%)	304 (33.5%)	0.99 (0.87–1.23)	49 (33.8%)	125 (27.9%)	1.14 (0.89–1.50)^b^
SAE Clavien Dindo III, No patients (%)	108 (12.4%)	129 (14.2%)	0.87 (0.69–1.11)	21 (14.5%)	46 (10.3%)	1.13 (0.90–2.29)^b^
SAE Clavien Dindo IV, No patients (%)	51 (5.9%)	69 (7.6%)	0.77 (0.54–1.09)	10 (6.9%)	26 (5.9%)	1.14 (0.56–2.32)^c^
SAE Clavien Dindo V, No patients (%)	15 (1.7%)	8 (0.9%)	1.96 (0.84–4.60)	2 (1.4%)	3 (0.7%)	1.30 (0.19–9.00)

a Adjusted for cardiovascular disease, COPD, insulin dependent diabetes, OR duration and type of surgery.

b Adjusted for insulin dependent diabetes, OR duration and type of surgery.

c Adjusted for cardiovascular disease, COPD, insulin dependent diabetes.

Analyses of attributive effects of individual interventions indicated that the attributive effect of hyperoxygenation was RR 0.95 (95% CI 0.70–1.33), GDFT RR 1.33 (95% CI: 1.00–1.75), normothermia RR 1.06 (95% CI: 0.85–1.33), wound irrigation RR 0.90 (95% CI: 0.67–1.20) and glucose control RR 0.97 (95% CI: 0.73–1.27). We did not find interactions between the interventions (p > 0.05). There was no dose response effect (Cochran Armitage test, p = 0.41). Analysis revealed no change in standard care, Online Appendix 10. We did not perform sensitivity analyses for the within-centre effect as major test assumptions were not met, Online Appendix 11.

Self-reported wound photos and questionnaires were analysed to determine underreporting of the primary outcome. Potentially 28 SSI were not reported with current follow up strategy. These were evenly spread across randomization groups, 15 in the intervention versus 12 in the control group. Details are provided in Online Appendix 12.

## Discussion

In patients undergoing abdominal surgery, the application of the EPO_2_CH bundle consisting of five evidence-based and individually effective interventions derived from recent guidelines on SSI prevention: intraoperative high fraction of inspired oxygen (FiO_2_ 0.80); Goal-Directed Fluid Therapy; active pre-, intra-, and postoperative warming; strict perioperative glucose control; and incisional wound irrigation with an aqueous antiseptic agent,^7^, ^8^, ^9^, ^10^ added to standard care in a high-income health care setting did not reduce the incidence of SSI. Analysis of postoperative health care provider questionnaires and process measures showed a large difference in provided care of each individual intervention of the bundle between the intervention and control group, confirming successful execution of the study protocol. Analyses of the attributive effect of the individual interventions indicated none of the interventions individually reduced the risk of SSI.

Three major evidence-based guidelines on SSI prevention recommend the use of these five interventions.^7^, ^8^, ^9^, ^10^ Each intervention individually is supported by evidence of benefit from a systematic review of RCTs.^7^, ^8^, ^9^, ^10^ It is therefore surprising that no reduction in SSI was observed when these interventions were successfully applied as a bundle. Despite being based on the best available evidence at the time, some of the interventions have become a topic of debate. The effect of the use of *hyperoxygenation* has been disputed as recent clinical studies were unable to replicate the previously observed beneficial effect.^17^, ^18^, ^19^ Similarly, *Goal-Directed Fluid Therapy* seems less effective than early meta-analyses have indicated.^20^, ^21^, ^22^, ^23^ The evidence for *wound irrigation* stems from older and relatively small studies that could not be replicated by a large RCT in 2016.^24^, ^25^, ^26^ A recent RCT on *temperature management* found that strict intraoperative temperature management aimed at 37.0 °C does not reduce SSI risk when compared to 35.5 °C.^27^ While recent meta-analyses continue to support the interventions included in EPO_2_CH, this may be driven by data that are less representative for current standards of care in a high-income health care setting.^23^^,^^28^^,^^29^ Perioperative care evolves continuously with innovations and over time, these changes may have modified the effect of the interventions and led to the incongruent results.

Another potential explanation for the observed lack of effect applicable to *temperature management* and *glucose control* may be that the interventions insufficiently contrasted with standard care. Because strict *glucose control* increases mortality rates in critically ill patients, we designed our glucose control algorithm defensively triggering insulin treatment only after blood glucose exceeded 10 mmol per litre.^30^ However, few patients (14.2% (123/869) in the intervention group) had blood glucose above this threshold and treatment was rarely triggered. As a result, no large effect on SSI can reasonably be expected. Similarly, *temperature management* under standard care in this high-income setting was more effective than expected. This may explain why the addition of pre- and post-operative warming provided no additional benefit on perioperative core temperature.

Effective implementation of evidence-based care programs has proven difficult in the past. Examples include failed implementation of the surgical safety checklist after initial striking results in RCTs and the coincidental namesake EPOCH that trialled implementation of an evidence-based care pathway for emergency abdominal surgery in the UK.^31^, ^32^, ^33^ Overall, compliance to the trial protocol was good and clear contrasts were observable between provided care in the intervention and control group. However, this is a pragmatic trial reflecting real-life practice and a complex multifaceted intervention. Each individual component is a risk to fail the overall cumulative adherence. Interventions that comprised of several steps in different phases of perioperative care such as glucose control and perioperative warming were particularly challenging. Imperfect execution of the interventions could have contributed to underestimation of the effect. Similarly, there was some contamination between groups as physicians applied interventions to some of the patients in the control group at their discretion. To account for these challenges and asses the effect of perfect execution of the intervention compared to standard care without any of the interventions we designed a strict per protocol definition to complement the intention to treat analysis. Inherently, this results in a sharp contrast in size between the intention to treat and per protocol population with fewer events and wider confidence intervals. The per protocol analysis indicated that better compliance, execution or less contamination would not have resulted in different results for the primary outcome. Similarly, there was no evidence for different results on other outcomes although confidence intervals were wide. Although statistically insignificant, the incidence of ICU admission and mortality was notably higher for intervention patients in the per-protocol analysis. This is likely a chance finding in an analysis with few events as the DSMB indicated no observed harm was relatable to the interventions.

The observed incidence of SSI was considerably higher than expected at the time of the study design. This is likely the result of a combination of factors including underestimation of the true SSI incidence by surveillance data and intensive monitoring during the study. Compared to planned inclusion, more participants (50.5%) were included at an academic hospital. Therefore, a large proportion of patients underwent complex, high risk or revision surgery with a higher risk of complications including SSI. Notably, the observed incidence and distribution in wound class is comparable to similar high quality RCTs^34^ on SSI in this population. Contaminated and dirty wounds are rare in elective surgery with advanced surgical techniques. Interestingly, the proportion of superficial SSI was relatively low. As the analysis from the wound photographs suggests, some superficial SSI may have remained unnoticed in case they emerged after routine outpatient follow up and the participant did not seek care, as no additional visit was scheduled at the end of follow up. However, when participants did not seek care, the SSI was likely of limited clinical relevance. Based on the photograph evaluation this occurred in 3.4% (28/822) of patients. Considering the density of hospitals in the Netherlands and the instructions to seek care in case of concerns, it is unlikely that clinically relevant SSI were treated elsewhere. Importantly, this phenomenon of seeking care and follow-up after discharge is unrelated to the intervention and distributed across the two groups. There where incidental cases of reoperation for other causes than SSI that occurred within the follow up. Due to the small numbers in relation to the overall study size any potential interference with the primary outcome from these cases was considered negligeable.

We have documented the design and execution of this trial in detail. This transparent and pragmatic approach ensures good external validity. Our meticulously predefined and peer reviewed statistical analysis plan has helped mitigate potential bias.^12^ Yet, there are several limitations to this study. Bowel preparation is uncommon in the Netherlands. Only 23 patients received chemical bowel preparation, equally distributed across the two groups, and there are no details available on mechanical bowel preparation. Weight based dosing was not routinely practiced during the study but considering the mean BMI in the population and the equal distribution across the two groups (mean, SD: 26.6, 4.70 and 26.4, 5.24 respectively) it is unlikely that this would have affected the results. When studying a bundled intervention, it is hard to ascertain which components are important to a potentially observed effect. Conversely, when one finds no effect, it is likely that none of the interventions are effective. Due to the limited contrast, we cannot draw firm conclusions on the effect of perioperative glucose control and normothermia compared to no intervention at all. However, this study does clearly demonstrate that a considerable proportion of patients experience hyperglycaemic episodes during and after surgery that warrant treatment. Almost half of these patients are not diabetic and would not have been identified without routine blood glucose control resulting in more than double the amount of identified (14.2% (123/869) versus 7.3% (65/908) and treated (13.6% (118/869) versus 5.2% (47/908)) hyperglycaemic episodes in the intervention group. Perioperative glucose control may still be effective with a lower target blood glucose level and strict execution of insulin administration.^35^ Normothermia is likely still effective when compared to hypothermic controls. However, for wound irrigation, GDFT, and FiO_2_, our findings cast doubt on their effectiveness in this setting. Although investigated in a bundle, this study would rank among the largest trials on the topic for each separate intervention. Given the recent guideline recommendations and efforts to implement these globally, these findings have important practice and policy implications. Resources should be allocated towards implementation of interventions that have been proven beyond any doubt such as hand preparation, surgical site preparation and adequate use of preoperative antibiotic prophylaxis. Considering the persistent high risk of SSI even in high resource settings, research into interventions that may help reduce this risk remains urgently needed. A promising area of interest is the patient’s microbiome and related personalised interventions, to attenuate these patient specific SSI risk factors.

In conclusion, in patients undergoing abdominal surgery no effect on surgical site infections was observed after adequately executed application of a focused care bundle based on recent SSI prevention guidelines added to standard care in a high-income health care setting.

## Contributors

The study was designed by MAB, MWH, MGWD & SWdJ. NW & QJB coordinated the trial. NW, QJB, WJB, LMP, JCGS and SWdJ ran the trial and collected data. NW and SWdJ verified the underlying data. NW conducted all statistical analyses under supervision of SWdJ and MGWD. NW and SWdJ prepared the first draft of the manuscript. All authors [NW, QJB, WJB, LMP, JCGS, BMFvdL, JABvdH, JPH, DJAS, OEvG, ERH, EBK, AD, LRCWvL, MGWD, MWH, MAB, SWdJ] have access to the data, contributed to the final draft of the manuscript, and had final responsibility to publish the manuscript.

## Data sharing statement

Data sharing of deidentified participant data is considered upon reasonable request after approval of a protocol and signing of a data transfer agreement through the corresponding author after publication of all secondary outcomes of this trial. Related documents are available through the corresponding author.

## Declaration of interests

MWH is Section Editor Pharmacology with Anaesthesia & Analgesia, Section Editor Anaesthesiology with J Clin Med and Editor Front Physiol. MWH received honoraria for advisory activities from IDD Pharma, Medical Developments and PAION and research grants from ZonMW and ESAIC. MAB reported receiving institutional grants from J&J/Ethicon and KCI/3M; and being a speaker and/or instructor (payment to institution) for J&J/Ethicon, 3M, BD, Gore, TelaBio, Medtronic, GD Medical, Smith&Nephew, Angiodynamics, Molnlycke. MAB takes part in the DSMB of the Extend Trial. NW, QJB, WJB, LMP, JCGS, BMFvdL, JABvdH, JPH, DJAS, OEvG, ERH, EBK, AD, LRCWvL, MGWD, SWdJ report no conflict of interest.

## Appendix A Supplementary data

Appendices 1–13
