# Supplementary material for: A care bundle added to standard care versus standard care for the prevention of surgical site infections after abdominal surgery (EPO2CH trial): a randomised, open label, pragmatic, superiority multicentre trial
Source: Lancet Reg Health Eur. 2025 Sep 17;58:101448. doi: 10.1016/j.lanepe.2025.101448 (PMC12478259; doi:10.1016/j.lanepe.2025.101448)
Supplement: Appendices 1–13 [file mmc1.docx]

**A care bundle added to standard care versus standard care for the prevention of surgical site infections after abdominal surgery (EPO_2_CH trial); a randomised, open label, pragmatic, superiority multicentre trial**

Niels Wolfhagen, Quirine J.J. Boldingh, Wouter J. Bom, Linda M. Posthuma, Jochem C.G. Scheijmans, Bart M.F. van der Leeuw, Joost A.B. van der Hoeven, Jens-Peter Hering, Dirk J.A. Sonneveld, Otto E. van Geffen, Eduard R. Hendriks, Ewoud B. Kluyver, Ahmet Demirkiran, Luc R.C.W. van Lonkhuijzen, Marcel G.W. Dijkgraaf, Markus W. Hollmann, Marja A. Boermeester, Stijn W. de Jonge

**Supplementary appendix**

Appendix 1: Preoperative surgical antibiotic prophylaxis

Appendix 2: Treatment of surgical site infections

Appendix 3: Provided care according to health care provider questionnaires

Appendix 4: Details regarding wound irrigation

Appendix 5: Predefined definitions for successful execution of interventions

Appendix 6: Compliance to the intervention protocol versus standard care including evaluation by process measures

Appendix 7: Details regarding intraoperative glucose measurement

Appendix 8: Details on confounder selection

Appendix 9: Details on serious adverse events

Appendix 10: Additional safety analysis

Appendix 11: Analysis of standard care

Appendix 12: Considerations regarding possible *within-centre* effect

Appendix 13: Results from self-reported wound photos and questionnaires

**Appendix 1:** **Preoperative surgical antibiotic prophylaxis**

Indicative selection of patients, based on available data records from electronic health record.

|  | Intervention group  (n = 723) | Control group  (n = 759) |
| --- | --- | --- |
| Amoxicilline/clavulanic acid | 1 (0·1%) | 0 (0·0%) |
| Amoxicilline/clavulanic acid and metrodinazol | 0 (0·0%) | 1 (0·1%) |
| Amoxicilline/clavulanic acid and gentamycin | 1 (0·1%) | 0 (0%) |
| Amoxicilline, metrodinazol and cefazolin | 2 (0·3%) | 0 (0%) |
| Amoxicilline and gentamycin | 1 (0·1%) | 0 (0%) |
| Cefazoline | 42 (5·8%) | 26 (3·4%) |
| Cefazoline, gentamycin and metrodinazol | 2 (0·3%) | 1 (0·1%) |
| Cefazoline and metrodinazol | 647 (89·5%) | 709 (93·4%) |
| Cefuroxim and metrodinazol | 0 (0%) | 1 (0·1%) |
| Ceftazidim and metrodinazol | 2 (0·3%) | 0 (0%) |
| Ceftriaxon and gentamycin | 3 (0·4%) | 2 (0·3%) |
| Ceftriaxon and metrodinazol | 0 (0%) | 1 (0·1%) |
| Clindamycine | 2 (0·3%) | 2 (0·3%) |
| Clindamycine and metrodinazol | 1 (0·1%) | 0 (0·0%) |
| Cotrimoxazol | 0 (0·0%) | 1 (0·1%) |
| Gentamycine and clindamycin | 12 (1·7%) | 8 (1·1%) |
| Gentamycine and metrodinazol | 2 (0·3%) | 0 (0·0%) |
| Gentamycine, vancomycin and clindamycin | 0 (0%) | 1 (0·1%) |
| Metrodinazol | 5 (0·7%) | 3 (0·4%) |
| *Typical dosages are cefazoline 2000 mg and metrodinazol 500 mg. | | |

**Appendix 2: Treatment of surgical site infections**

**Treatment details of superficial surgical site infections**

|  | Intervention group  (n = 52) | Control group  (n = 52) |
| --- | --- | --- |
| No intervention | 1 (1·9%) | 6 (11·8%) |
| Antibiotics | 23 (44·2%) | 16 (31·4%) |
| Opening of surgical wound | 35 (67·3%) | 32 (62·7%) |
| Radiological intervention | 0 (0%) | 1 (2%) |
| Surgical intervention | 0 (0%) | 0 (0%) |
| Vacuum wound dressing | 0 (0%) | 1 (2·0%) |
| Other | 2 (3·8%) | 13 (5·9%) |
| *Some wound infections were multimodally treated | | |

**Treatment details of deep surgical site infections including anastomotic leakage**

|  | Intervention group  (n = 107) | Control group  (n = 119) |
| --- | --- | --- |
| No intervention | 1 (0·9%) | 0 (0%) |
| Antibiotics | 88 (82·2%) | 93 (78·2%) |
| Opening of surgical wound | 36 (33·6%) | 28 (23·5%) |
| Radiological intervention | 59 (55·1%) | 55 (46·2%) |
| Surgical intervention | 36 (33·6%) | 49 (41·2%) |
| Vacuum wound dressing | 13 (12·1%) | 12 (10·1%) |
| Other | 6 (5·6%) | 8 (6·7%) |
| *Some wound infections were multimodally treated | | |

**Appendix 3: Provided care according to health care provider questionnaires**

|  | Intervention group  (n = 869) | Control group  (n = 908) |
| --- | --- | --- |
| **Normothermia (%)** |  |  |
| Intra-operative warming (%) | 679 (88·0%) | 708 (88·2%) |
| Active pre- or postoperative warming (%) | 652 (84·5%) | 165 (20·5%) |
| Missing | 97 | 105 |
| **Goal Directed Fluid Therapy (%)** | 669 (84·8%) | 167 (21·2%) |
| Missing | 80 | 120 |
| **Hyperoxygenation (%)*** | 659 (77·0%) | 43 (4·8%) |
| Missing | 13 | 14 |
| **Normoglycemia (%)**** |  |  |
| Intraoperative glucose measurement (%) | 603 (93·3%) | 157 (21·0%) |
| Missing | 82 | 120 |
| **Wound irrigation (%)** | 711 (89·8%) | 141 (17·0%) |
| Missing | 77 | 78 |
| *based on process parameters instead of postoperative questionnaires  **excluding diabetic patients | | |

**Appendix 4: Details regarding wound irrigation**

Data on wound irrigation was obtained from postoperative surveys. Missing data concerns invalid or omitting postoperative survey by the surgeon.

|  | Intervention group  (n = 869) | Control group  (n = 908) |
| --- | --- | --- |
| No wound irrigation | 81 (9·3%) | 689 (75·9%) |
| Aqueous povidone-iodide | 445 (51·2%) | 98 (10·8%) |
| Aqueous chlorhexidine | 254 (29·2%) | 28 (3·1%) |
| NaCl | 12 (1·4%) | 15 (1·7%) |
| Missing | 77 (8·9%) | 78 (8·6%) |
|  | | |

**Appendix 5: Predefined definitions for successful execution of interventions**

| Intervention | Definition for intervention group | Definition for control group |
| --- | --- | --- |
| Hyperoxygenation | FiO_2_ of 0·80 ± 0·05 for at least 75 percent of the ventilation time | FiO_2_ of smaller than 0·40 ± 0·05 for at least 75 percent of the ventilation time |
| Normothermia | Temperature above 36·5 ◦C ± 0·5 ◦C, from intubation until one hour postoperatively | All participants meet definition due to pragmatic character of trial |
| Normoglycemia | blood glucose levels < 10 mmol L-1 or > 10 mmol L-1 with adequate treatment, measured every hour during surgery or at least twice for procedures lasting more than two hours, and once at the recovery, at day one and day two postoperatively | All participants meet definition due to pragmatic character of trial |
| Goal-directed fluid therapy | Goal-directed fluid therapy as reported by anaesthetist in postoperative survey· Method of GDFT is at the discretion of treating physician· | No goal-directed fluid therapy as reported by anaesthetist in postoperative survey· |
| Wound irrigation | No wound irrigation as reported by surgeon in postoperative survey· Method of wound irrigation is at the discretion of treating physician· | No wound irrigation as reported by surgeon in postoperative survey· |

**Appendix 6: Compliance to the intervention protocol versus standard care including evaluation by process measures**

| Intervention | Intervention group  (n = 869) | Control group  (n = 908) |
| --- | --- | --- |
| **Normothermia (%)** # |  |  |
| Yes | 530 (61·0%) | 449 (49·4%) |
| No | 307 (35·3%) | 422 (46·5%) |
| Missing (%) | 32 (3·7%) | 37 (4·1%) |
| Mean minimum temperature, °C | 35·5 (1·1) | 35·6 (1·4) |
| Missing | 153 (16·9%) | 152 (17·5%) |
| Mean temperature PACU, °C | 36·8 (0·6) | 36·6 (0·6) |
| Missing | 212 (24·4%) | 270 (29·7%) |
| **Goal Directed Fluid Therapy (%)** * |  |  |
| Yes | 669 (77·0%) | 167 (18·4%) |
| No | 120 (13·8%) | 621 (68·4%) |
| Missing (%) | 80 (9·2%) | 120 (13·2%) |
| **Hyperoxygenation (%)** # |  |  |
| Yes | 659 (75·8%) | 43 (4·7%) |
| No | 197 (22·7%) | 851 (93·7%) |
| Missing (%) | 13 (1·5%) | 14 (1·5%) |
| Fraction of inspired oxygen (%) | 74·4 (10·3) | 48·6 (10·5 |
| **Normoglycemia (%)** # |  |  |
| Yes | 506 (58·2%) | 50 (5·5%) |
| No | 362 (41·7%) | 857 (94·4%) |
| Missing (%) | 1 (0·1%) | 1 (0·1%) |
| Intraoperative glucose > 10 mmol per litre (%) | 123 (14·2%) | 65 (7·2%) |
| Intraoperative insulin administration (%) | 118 (13·6%) | 47 (5·2%) |
| **Wound irrigation (%)** * |  |  |
| Yes | 711 (81·8%) | 141 (15·5%) |
| No | 81 (9·3%) | 689 (75·9%) |
| Missing (%) | 77 (8·9%) | 78 (8·6%) |
| * provided care evaluated by postoperative questionnaire  # evaluated by postoperative questionnaire and additional process measures. Normothermia was evaluated by minute-to-minute temperature measurements. Hyperoxygenation by minute-to-minute administered intraoperative FiO2. Normoglycemia by glucose measurements and insulin administration registered in the electronic health record. | | |

**Appendix 7: Details regarding intraoperative glucose measurement**

Predefined definitions for successful execution of normoglycemia were multimodally determined. If either the postoperative survey or data from the electronic health record was considered successful, the overall executions was considered successful.

Intraoperative glucose was available from the electronic health record for 695 patients in total. Glucose measurements and insulin administration of the other participants were collected through postoperative surveys, questionnaires or manual.

|  | Intervention group  (n = 463) | Control group  (n = 232) |
| --- | --- | --- |
| Number of measurements, mean (sd) | 2·55 (1·60) | 1·81 (1·18) |
| Mean glucose, mean (sd) | 7·42 (1·88) | 7·74 (2·08) |

**Baseline characteristics and SSI incidence of participants with intraoperative glucose above 10 mmol**

|  | Intervention group  (n = 123) | Control group  ( n = 65) |
| --- | --- | --- |
| Sex, male | 61 (49·6%) | 29 (46·6%) |
| Age, years | 66·2 (10·9) | 66·2 (11·0) |
| BMI, kg/m^2^ | 27·7 (5·24) | 26·9 (7·04) |
| ASA physical status score |  |  |
| ASA I | 6 (4·9%) | 3 (4·6%) |
| ASA II | 65 (52·8%) | 33 (50·8%) |
| ASA III | 50 (40·7%) | 27 (41·5%) |
| ASA IV | 2 (1·6%) | 2 (3·1%) |
| Smoking | 61 (49·6%) | 34 (52·3%) |
| Diabetes mellitus | 60 (48·8%) | 33 (50·8%) |
| Chronic Obstructive Pulmonary Disease | 11 (8·9%) | 9 (13·8%) |
| History of abdominal surgery | 70 (56·9%) | 333 (50·8%) |
| Cardiovascular disease excl· hypertension | 25 (20·3%) | 16 (26·2%) |
| Type of hospital |  |  |
| Academic | 81 (65·9%) | 57 (87·7%) |
| Top-clinical | 34 (27·6%) | 2 (3·1%) |
| General | 8 (6·5%) | 6 (9·2%) |
| Indication for surgery |  |  |
| Benign | 31 (25·2%) | 16 (24·6%) |
| Malignancy | 92 (74·8%) | 49 (75·4%) |
| Surgery type |  |  |
| General surgery | 18 (14·6%) | 6 (9·2%) |
| Upper gastrointestinal surgery | 7 (5·70%) | 0 (0%) |
| Hepato-pancreatico-biliary | 33 (26·8%) | 32 (49·2%) |
| Colorectal surgery | 49 (39·8%) | 17 (26·2%) |
| Gynecologic surgery | 16 (13·0%) | 10 (15·4%) |
| SSI | 36 (29·3%) | 17 (26·2%) |
| Continuous variables are expressed as mean (SD), discrete variables are expressed as number (%) | | |

**Baseline characteristics of participants with [insulin]dependent diabetes mellitus**

|  | [insulin]dependent diabetes mellitus (n = 267) | Not [insulin]dependent diabetes mellitus  (n = 1510) |
| --- | --- | --- |
| Sex, male | 151 (56·2%) | 630 (41·7%) |
| Age, years | 68·7 (9·14) | 62·5 (13·0) |
| BMI, kg/m^2^ | 28·3 (5·79) | 26·1 (4·76) |
| ASA physical status score |  |  |
| ASA I | 0 (0%) | 199 (13·2%) |
| ASA II | 150 (56·2%) | 997 (66·3%) |
| ASA III | 112 (41·9%) | 298 (19·8%) |
| ASA IV | 0 (0%) | 9 (0·60%) |
| Smoking | 130 (48·7%) | 845 (46·3%) |
| Chronic Obstructive Pulmonary Disease | 35 (13·1%) | 98 (6·50%) |
| History of abdominal surgery | 138 (51·7%) | 717 (47·5%) |
| Cardiovascular disease excl· hypertension | 75 (28·1%) | 224 (14·8%) |
| Type of hospital |  |  |
| Academic | 145 (54·,%) | 752 (49·8%) |
| Top-clinical | 75 (28·1%) | 425 (28·1%) |
| General | 47 (17·6%) | 333 (22·1%) |
| Indication for surgery |  |  |
| Benign | 64 (24·0%) | 394 (26·1%) |
| Malignancy | 203 (76·0%) | 1115 (73·8%) |
| Surgery type |  |  |
| General surgery | 37 (13·9%) | 199 (13·2%) |
| Upper gastrointestinal surgery | 12 (4·50%) | 35 (2·30%) |
| Hepato-pancreatico-biliary | 67 (25·1%) | 206 (13·6%) |
| Colorectal surgery | 128 (47·9%) | 854 (56·6%) |
| Gynecologic surgery | 23 (8·60%) | 216 (14·3%) |
| SSI | 56 (21·0%) | 276 (18·3%) |
| Continuous variables are expressed as mean (SD), discrete variables are expressed as number (%) | | |

**Appendix 8: Details on confounder selection**

We considered confounders based on confounder selection by VanderWeele and Shpitser.^1,2^ We considered preoperative variables that potentially influence the observed effect, even after randomization. Furthermore we also considered procedure duration as a proxy-variable for the complexity of the procedure. Statistical variable selection was based on backward selection. Potential confounders eligible for consideration were preoperative body mass index, insulin dependent diabetes mellitus, known cardiovascular comorbidities [besides hypertension], known chronic obstructive pulmonary disease, age, sex, ASA classification, smoking, previous abdominal surgery, type of surgery, surgery indication and duration of surgery.

Confounders that passed all [statistical] criteria and were included in the model were cardiovascular comorbidities, chronic obstructive pulmonary disease, insulin dependent diabetes mellitus, duration of surgery and type of surgery.**Appendix 9: Details on serious adverse events**

Follow MedDRA serious adverse events (SAE) were categorized on the level of System Organ Class. As these are not mutually exclusive SAEs can be categorized in multiple classes. Dominant classes are reported, for example an anastomotic leakage is primarily considered a ‘Gastrointestinal disorder.’ Secondarily it is also considered an infection and can also be considered “Infection.” The table below reports all SAE in the dominant class.

|  | Intervention group  (n = 531) | Control group  (n = 543) |
| --- | --- | --- |
| Blood and lymphatic disorders | 18 (3·4%) | 20 (3·7%) |
| Cardiac disorders | 25 (4·7%) | 25 (4·6%) |
| Congenital, familial and genetic disorders | 0 (0·0%) | 0 (0·0%) |
| Ear and labyrinth disorders | 1 (0·2%) | 0 (0·0%) |
| Endocrine disorders | 7 (1·3%) | 7 (1·3%) |
| Eye disorders | 2 (0·4%) | 0 (0·0%) |
| Gastrointestinal disorders | 161 (30·3%) | 181 (33·3%) |
| General disorders and administration site conditions | 0 (0·0%) | 0 (0·0%) |
| Hepatobiliary disorders | 25 (4·7%) | 31 (5·7%) |
| Immune system disorders | 1 (0·2%) | 0 (0·0%) |
| Infections and infestations | 118 (22·2%) | 113 (20·8%) |
| Injury, poisoning and procedural complications | 1 (0·2%) | 0 (0·0%) |
| Investigations | 0 (0·0%) | 0 (0·0%) |
| Metabolism and nutrition disorders | 4 (0·8%) | 8 (1·5%) |
| Musculoskeletal and connective disorders | 0 (0·0%) | 0 (0·0%) |
| Neoplasm benign, malignant and unspecified | 3 (0·6%) | 1 (0·2%) |
| Nervous system disorders | 4 (0·8%) | 1 (0·2%) |
| Pregnancy, puerperium and perinatal conditions | 0 (0·0%) | 0 (0·0%) |
| Product issues | 1 (0·2%) | 0 (0·0%) |
| Psychiatric disorders | 14 (2·6%) | 11 (2·0%) |
| Renal and urinary disorders | 28 (5·3%) | 35 (6·4%) |
| Reproductive system and breast disorders | 0 (0·0%) | 0 (0·0%) |
| Respiratory, thoracic and mediastinal disorders | 52 (9·8%) | 67 (12·3%) |
| Skin and subcutaneous tissue disorders | 0 (0·0%) | 0 (0·0%) |
| Social circumstances | 0 (0·0%) | 1 (0·2%) |
| Surgical and medical procedures | 7 (1·3%) | 4 (0·7%) |
| Vascular disorders | 58 (10·9%) | 38 (7·0%) |

**Appendix 10: Additional safety analysis**

The *safety* population includes all trial participants that received the allocated intervention. It is a larger population compared to the *intention-to-treat* population as participants that were postoperatively excluded and replaced are included in the safety population as the participants received any intervention, depending on the randomisation group.

**Safety analysis in safety population based on randomization group**

| Characteristic | Intervention group  (n =911) | Control group  (n =959) | Relative Risk (95% CI) safety population (n =1870) |
| --- | --- | --- | --- |
| Mortality 30 days | 15 (1·6%) | 10 (1·0%) | 1·59 (0·72 – 3·51) |
| Length of stay, median (IQR) | 6 (5) | 6 (5) | 1·00 (0·96 – 1·03) |
| ICU admission – yes/no | 84 (9·2%) | 94 (9·8%) | 0·94 (0·71- 1·24) |
| Readmissions – yes/no | 89 (9·8%) | 80 (8·3%) | 1·17 (0·88 - 1·56) |
| Serious Adverse Events, No patients (%) | 298 (33·2%) | 316 (33·3%) | 1·00 (0·88 - 1·13) |
| Serious Adverse Events requiring intervention, No patients (%) | 112 (12·3%) | 134 14·0%) | 0·88 (0·70 - 1·11) |

**Appendix 11: Analysis of standard care**

Aim: The EPO_2_CH trial concerns a pragmatic trial which compares the EPO_2_CH bundle with standard care· We aimed to determine if the standard care changed over course of the trial. The participants (908) in the control group were grouped in 100 consecutive procedures. The *final* two groups (n = 100 and n = 8) were merged into one group (n=108). Dichotomous variables were analysed using Pearson’s Chi^2^ test. If these were statistical significant (p < 0·05) an additional Pearson’s correlation test was performed to investigate the correlation. Positive correlations of > 0·30 were considered significant over time. Continuous variables were analysed using ANOVA.

**Analysis of standard care over time**

| Intervention of EPO_2_CH bundle | p-value | Pearsons’ correlation  [if appropriate] |
| --- | --- | --- |
| Dichotomous variables |  |  |
| Active warming upon entering the OR | 0·07 | n.a. |
| Active warming during surgery | *0·02* | -0·34 |
| Active warming on PACU | 0·11 | n.a. |
| Hyperoxgenation conform protocol intervention group | 0·33 | n.a. |
| Measurement of intraoperative glucose | 0·19 | n.a. |
| Wound irrigation | 0·31 | n.a. |
| Goal directed fluid therapy | *0·02* | 0·04 |
| Continuous variables |  |  |
| Average intraoperative FiO_2_ | *0·004* | No trend towards intervention group |
| Average intraoperative temperature | *0·01* | No trend towards intervention group |
| *Abbreviations: FiO_2_; Fraction of inspired oxygen, NA; not applicable, OR; operation room, PACU; Postoperative Anaesthesia Care Unit* | | |

**Appendix 12: Considerations regarding possible *within-centre* effect**

As described in the statistical analysis plan, there may be a potential within-centre effect of the intervention. Accounting for this effect may increase power. While writing the study-protocol we specified assumptions and evaluated these after finishing the trial. When analysing the results we concluded that multiple of these assumptions were not met. Especially the assumption assuming a consistent number of participants per day seems troublesome as it is not consistent. The mean number of participants per day is around 2·34 while the standard deviation of de number is 1·35. These numbers exclude days on which no surgery was performed. Including these days will lead to much larger variation.

**Appendix 13: Results from self-reported wound photos and questionnaires**

From the intention to treat analysis total of 822 patients returned wound photos at 10 days, 30 days or both. Following the primary outcome 126 suffered an SSI. After assessment of the wound photographs possibly 28 additional SSI (3·4% (28/822)) were identified of which 15 (3·6% (15/412)) were in the intervention group and 13 (3·5% (13/410)) in the control group. All but two SSI, both in the intervention group, were, based on the wound photographs not deemed as requiring addition treatment and therefore not clinically relevant. It must be noted that assessment of wound photographs is challenging as no symptoms were not reported along the wound photographs.

**References**

1· VanderWeele TJ· Principles of confounder selection· Eur J Epidemiol 2019;34(3):211-219· DOI: 10·1007/s10654-019-00494-6·

2· VanderWeele TJ, Shpitser I· A new criterion for confounder selection· Biometrics 2011;67(4):1406-13· DOI: 10·1111/j·1541-0420·2011·01619·x·
